# Recent progress toward the asymmetric synthesis of carbon-substituted piperazine pharmacophores and oxidative related heterocycles

**DOI:** 10.1039/d0md00053a

**Published:** 2020-05-22

**Authors:** Plato A. Magriotis

**Affiliations:** a Department of Pharmacy , Laboratory of Medicinal Chemistry , University of Patras , Rio26504 , Greece . Email: pmagriotis@upatras.gr

## Abstract

The important requirement for approval of a new drug, in case it happens to be chiral, is that both enantiomers of the drug should be studied in detail, which has led synthetic organic and medicinal chemists to focus their attention on the development of new methods for asymmetric synthesis especially of relevant saturated N-heterocycles. On the other hand, the piperazine ring, besides defining a major class of saturated N-heterocycles, has been classified as a privileged structure in medicinal chemistry, since it is more than frequently found in biologically active compounds including several marketed blockbuster drugs such as Glivec (imatinib) and Viagra (sildenafil). Indeed, 13 of the 200 best-selling small molecule drugs in 2012 contained a piperazine ring. Nevertheless, analysis of the piperazine substitution pattern reveals a lack of structural diversity, with almost every single drug in this category (83%) containing a substituent at both the N1- and N4-positions compared to a few drugs having a substituent at any other position (C2, C3, C5, and C6). Significant chemical space that is closely related to that known to be biologically relevant, therefore, remains unexplored. In order to explore this chemical space, efficient and asymmetric syntheses of carbon-substituted piperazines and related heterocycles must be designed and developed. Initial, recent efforts toward the implementation of this particular target are in fact the subject of this review.

## Abstract 2

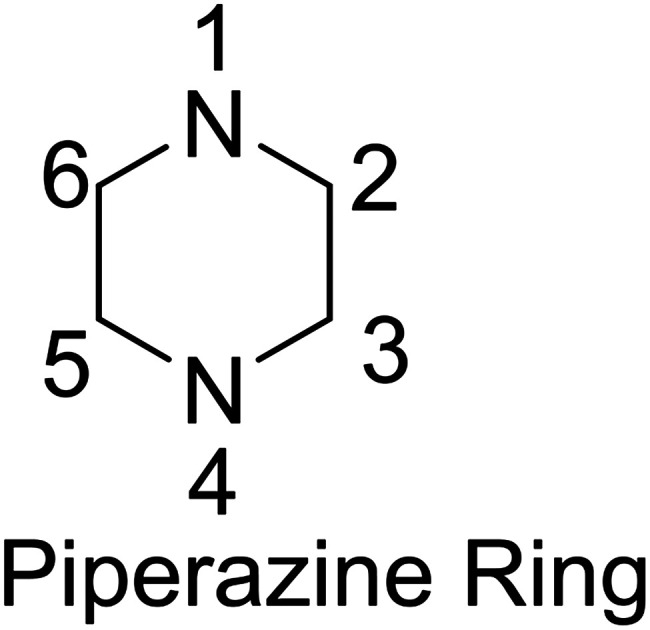
The piperazine drugs are mostly N-substituted compared to only a few C-substituted drugs. To explore this unknown chemical space, asymmetric syntheses of C-substituted piperazines is the subject of this review.

## Introduction

Heterocyclic small molecules are of exceedingly high importance in medicinal chemistry and drug discovery. Recently, the focus has shifted from purely aromatic N-heterocycles to substituted saturated N-heterocycles that have been indicated as the most valuable scaffolds for the development of new pharmaceuticals.^1^ Naturally, the latter entail a higher number of stereogenic centers. Consequently, new methods that allow practical access to these scaffolds with both absolute and relative stereocontrol are of very high and continuous interest. Furthermore, the important requirement for approval of a new drug, in case it happens to be chiral, is that both enantiomers of the drug should be studied in detail,^2^ which has led synthetic organic and medicinal chemists to focus their attention on the development of new methods for asymmetric syntheses especially of relevant saturated N-heterocycles.

On the other hand, the piperazine ring, besides defining a major class of saturated N-heterocycles, has been classified as a privileged structure in medicinal chemistry,^3^ since it is more than frequently found in biologically active compounds including several marketed blockbuster drugs such as Glivec (imatinib) and Viagra (sildenafil).^4^ Actually, an analysis of all U.S. FDA approved small molecule drugs found that 21% contained saturated 6-membered N-heterocycles with an additional heteroatom (N, piperazines; O, morpholines; S, thiomorpholines).^5^ Indeed, 13 of the 200 best-selling small molecule drugs in 2012 contain a piperazine ring.^6^ In the vast majority of these molecules, however, the piperazine ring is not substituted on any of its carbon atoms. Specifically, analysis of the piperazine substitution pattern reveals a lack of structural diversity, with almost every single drug in this category (83%) containing a substituent at both the N1- and N4-positions compared to a few drugs having a substituent at any other position (C2, C3, C5, and C6).^5^

Significant chemical space that is closely related to that known to be biologically relevant, therefore, remains unexplored. In order to explore this chemical space, efficient and asymmetric syntheses of carbon-substituted piperazines and related heterocycles must be designed and developed.^4^,^5^ Initial, recent efforts toward the implementation of this particular target are in fact the subject of this review since recent, elegant racemic approaches to piperazine synthesis have been properly reviewed.^7^

**Fig. 1:**
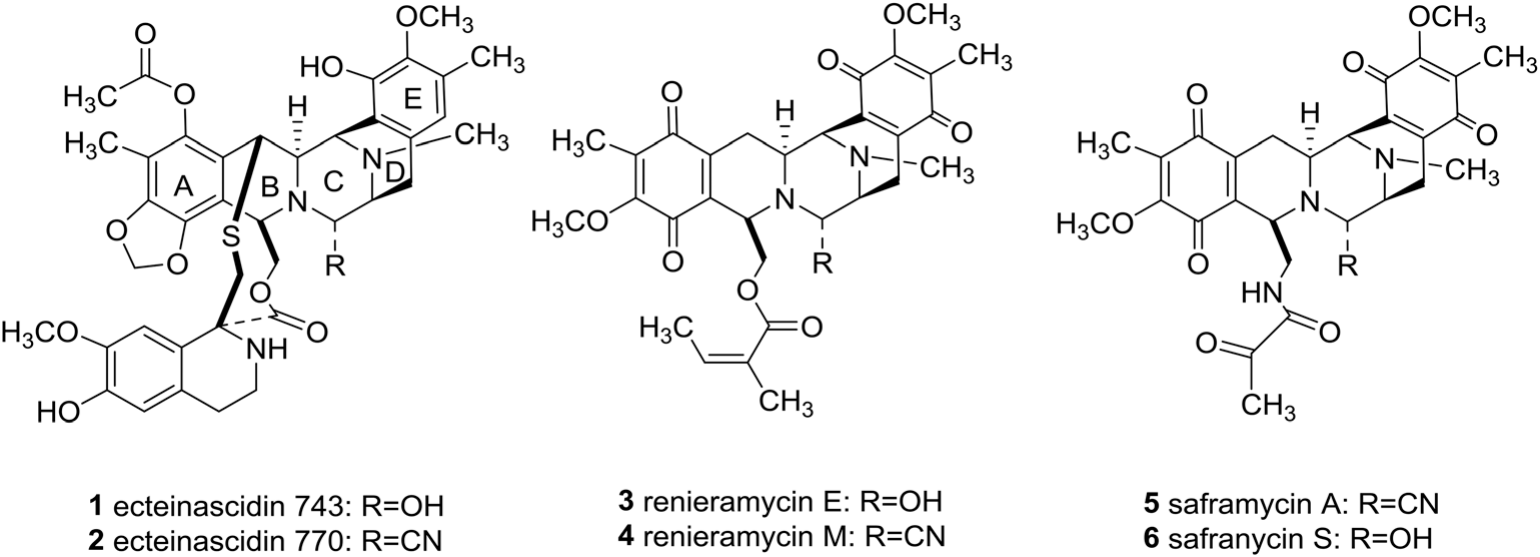
Marine bistetrahydroisoquinoline alkaloids.

Since piperazine derivatives have been reported to elicit a broad spectrum of pharmacological activities including antidepressant, anticancer, anthelmintic, antibacterial, antifungal, antimycobacterial, antimalarial, antituberculant, anticonvulsant,4*a* and anti-AIDS,^8^ one can easily comprehend that the sky will be the limit, as far as novel drug development is concerned, once asymmetric syntheses of this substituted heterocycle will be fully developed.^4^,^9^ Importantly, marine bis-tetrahydroisoquinoline alkaloids, including ecteinascidins (**1** and **2**), renieramycins (**3** and **4**), and saframycins (**5** and **6**, Fig. 1), besides sharing a densely functionalized common pentacyclic scaffold (A–E rings), they also feature a central C-polysubstituted piperazine as in the ecteinascidins (**1** and **2**, Fig. 1, ring C).

**Fig. 2:**
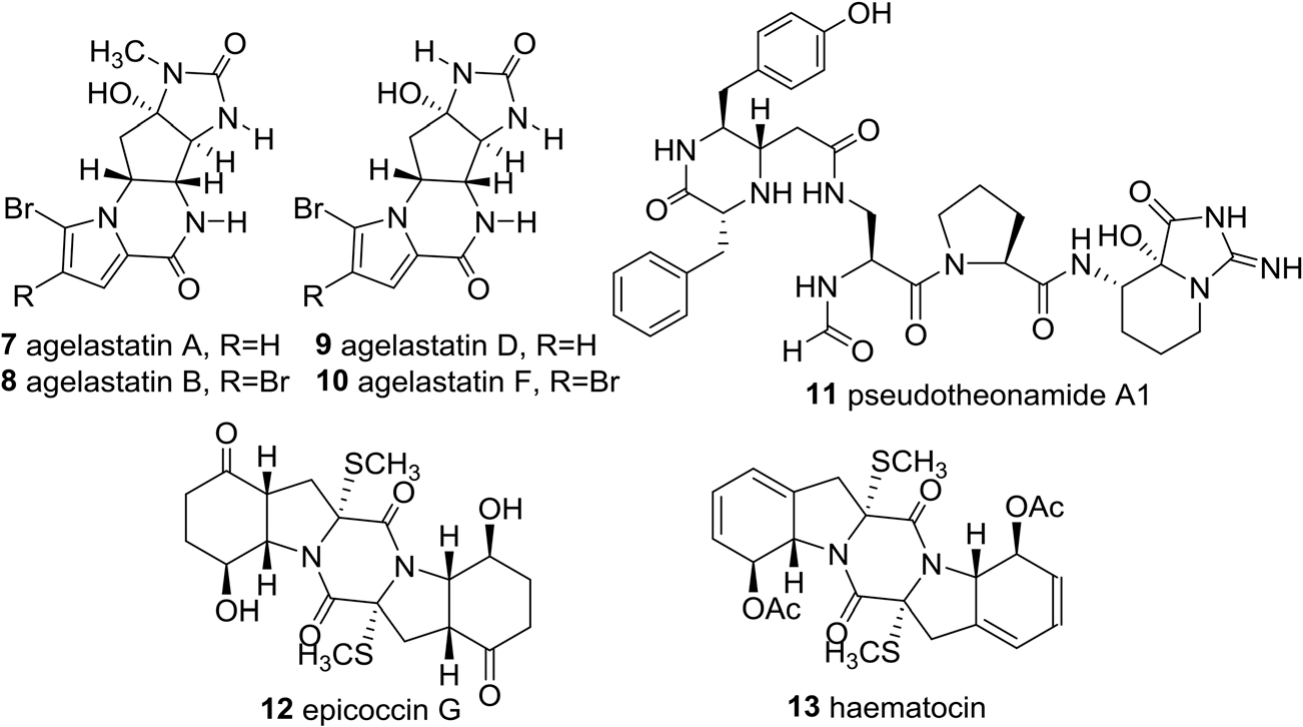
2-Oxopiperazine and 2,5-diketopiperazine containing natural products.

Significantly, the unique marine natural product ecteinascidin-743 (**1**, Et-743 also known as trabectedin or Yondelis™), isolated from the Caribbean tunicate *Ecteinascidia turbinate*,^10^ is a commercially available drug against soft-tissue sarcoma approved by the European Commission (EMEA) in 2007^11^ and the U.S. Food and Drug Administration (FDA) in 2015,^12^ and is also undergoing clinical trials for breast, prostate, ovarian, and pediatric sarcomas in other countries, due to its extraordinarily potent cytotoxicity activities against several rodent tumors and human tumor xenografts with *in vitro* IC_50_ values in the 0.1–1 ng mL^–1^ range.^13^ The complex molecular architecture, the remarkable biological activity, and the restricted natural availability (1.0 g from about 1.0 ton of tunicate) made Et-743 an exceedingly attractive target for total synthesis, and eight synthetic approaches to the stereochemically complex antitumor drug Et-743 completed between 1996 and 2015 have been reviewed.13b,^14^ Additionally, a convergent formal synthesis and a scalable total synthesis of Et-743 appeared in the literature in 2019.^15^ 2-Oxopiperazines (piperazinones) and 2,5-diketopiperazines are heterocycles related to piperazine by oxidation and they themselves represent key structural units of a great number of natural products such as agelastatins (**7–10**), pseudotheonamide A_1_ (**11**), and epicoccin G (**12**), as well as haematocin (**13**), respectively (Fig. 2).^16^

## Catalytic asymmetric syntheses of carbon-substituted piperazines and related heterocycles

### A. Catalytic enantioselective approaches

**Scheme 1:**
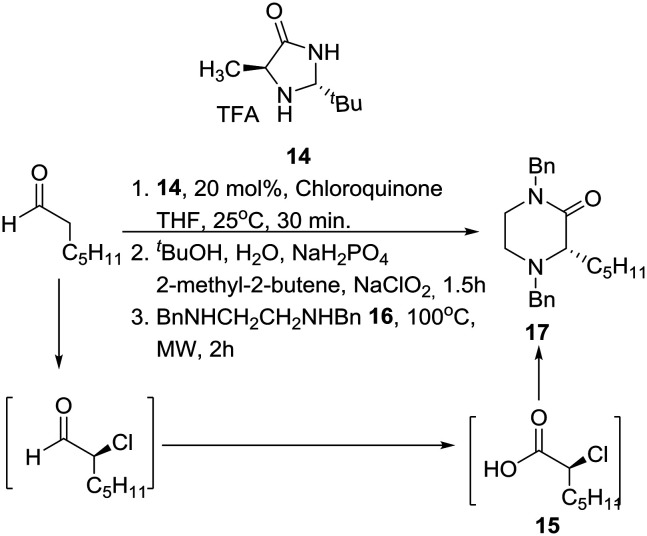
Kokotos' catalytic asymmetric synthesis of 2-oxopiperazines.

Interestingly, SAR studies on piperazinone derivatives as a novel class of potent HCV NS4B inhibitors have been reported,^17^ and a catalytic enantioselective synthesis of piperazinones from aldehydes was recently reported, entailing a four-step reaction sequence, not requiring any intermediate purification, and employing MacMillan's third-generation catalyst **14** and chloroquinone as the chlorinating reagent.^18^ Accordingly, mild catalytic asymmetric α-chlorination of heptanal was coupled with Pinnick oxidation leading to homochiral α-chloroheptanoic acid **15**. Nucleophilic substitution of the latter by *N*,*N*′-dibenzylethylenediamine (**16**) furnished the desired protected 2-oxopiperazine **17** in good overall yield under specific conditions (Scheme 1).

**Scheme 2:**
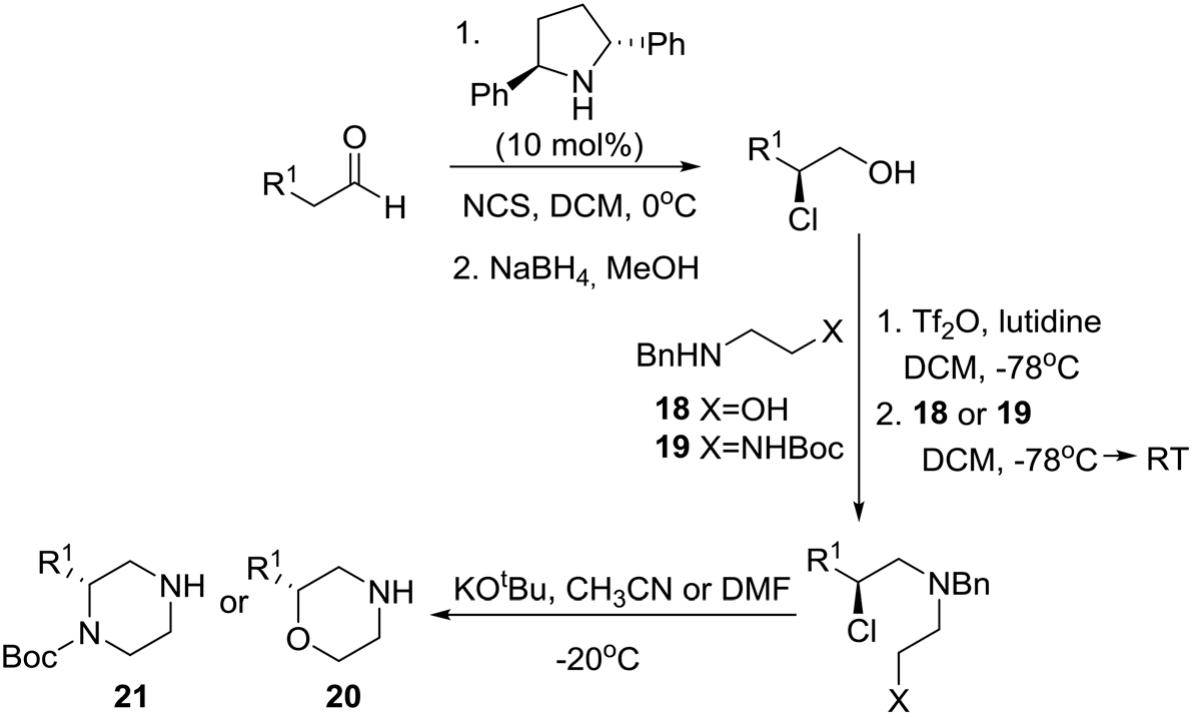
Lindsley's catalytic asymmetric syntheses of morpholines and piperazines.

A few years prior to this advance, a general, enantioselective synthesis of protected morpholines and piperazines was disclosed by Lindsley and O'Reilly based on Jørgensen's catalytic enantioselective α-chlorination of aldehydes followed by reduction to their corresponding 2-chloro alcohols (Scheme 2).^19^ Thus, homochiral-pyrrolidine catalyzed α-chlorination of aliphatic aldehydes followed by reduction to their corresponding 2-chloro alcohols, conversion to their respective triflates, chemoselective displacement with ambident nucleophiles **18** or **19**, and finally internal S_N_2 reaction of the chloride with inversion of configuration provided the desired morpholines **20** and piperazines **21**, respectively.

**Scheme 3:**
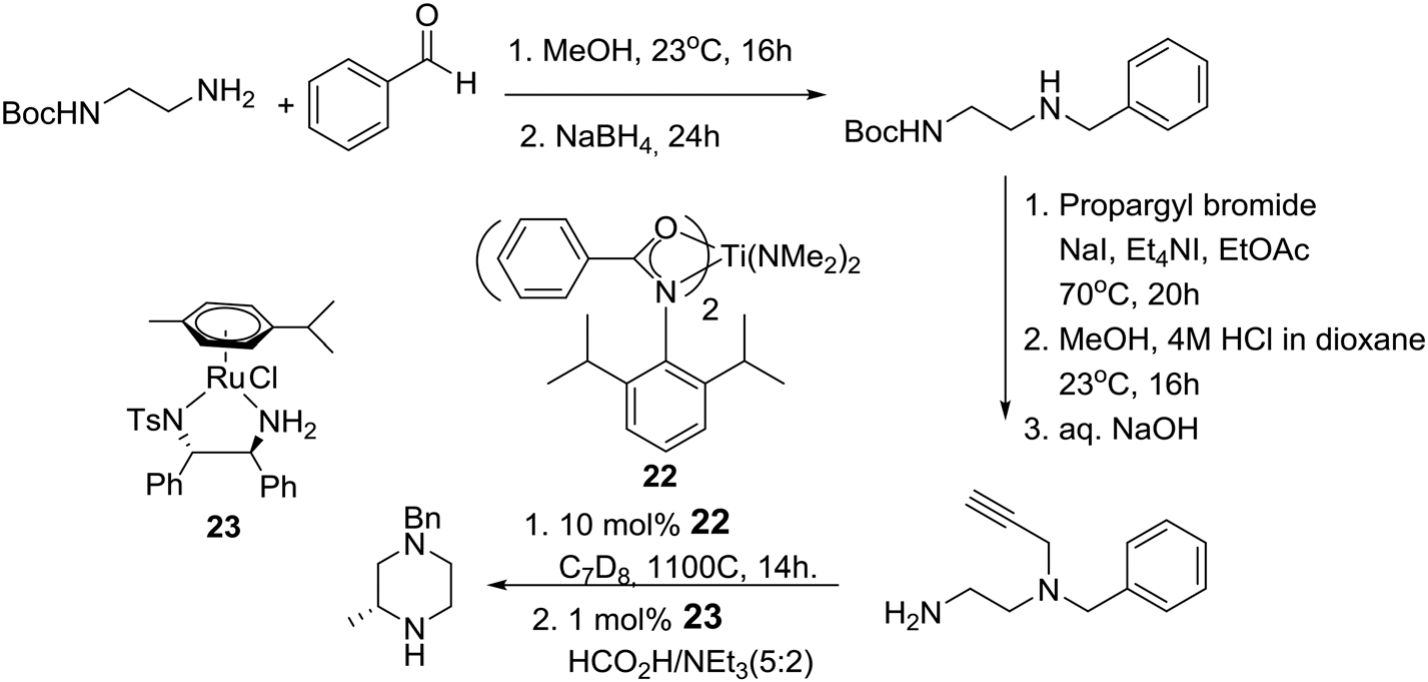
Schafer's catalytic asymmetric synthesis of piperazines.

A little later, the group of Laurel Schafer and coworkers in the U.S., after having developed a catalytic asymmetric synthesis of morpholines, used mechanistic insights from this process to realize a catalytic enantioselective synthesis of piperazines based on tandem hydroamination and asymmetric transfer hydrogenation reactions employing catalysts **22** and **23**, respectively, as described in Scheme 3.^20^

On the other hand, palladium-catalyzed asymmetric tandem allylic substitution of (*Z*)-1,4-bis(isopropoxycarbonyloxy)-2-butene (**24**) with the nucleophile 1,2-bis-(benzylamino)ethane (**16**), employing 2-(phosphinophenyl)pyridine (**25**) as a chiral ligand, furnished optically active 1,4-dibenzyl-2-vinylpiperazine (**26**) in 88% yield and 86% ee as described in eqn (1).^21^1
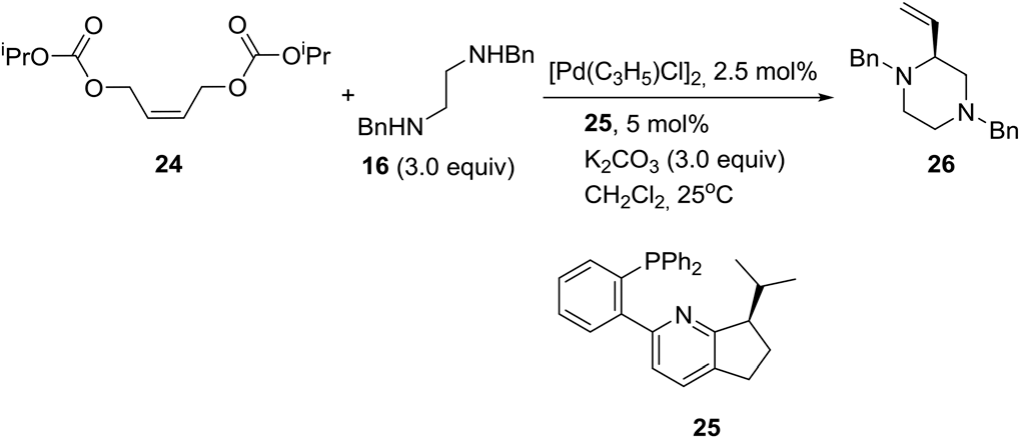



Reducing the unprecedented chemical space regarding carbon-substitution on piperazines, elucidated in the Introduction section, the group of Professor Stoltz in the U.S. reported the catalytic enantioselective synthesis of α-tertiary piperazin-2-ones and thereby piperazines by employing catalytic asymmetric allylation. Thus, the asymmetric palladium-catalyzed decarboxylative allylic alkylation of differentially N-protected piperazin-2-ones allowed the synthesis of a variety of highly enantioenriched tertiary piperazin-2-ones (eqn (2)). Deprotection and reduction afforded the corresponding tertiary piperazines, which were employed for the synthesis of medicinally important analogues such as imatinib analogues.^22^ Thus, treatment of α-tertiary piperazinone ester **27** with tris(4,4′-methoxydibenzylideneacetone)-dipalladium(0) ([pd_2_(pmdba)_3_]) at 5 mol% loading and (*S*)-(CF_3_)_3_-*t*-BuPHOX ligand **28** at 12.5 mol% loading in a 0.014 M solution of warm toluene produced the desired α-tertiary allylated product **29** in 77% yield and 96% ee (eqn (2)).2
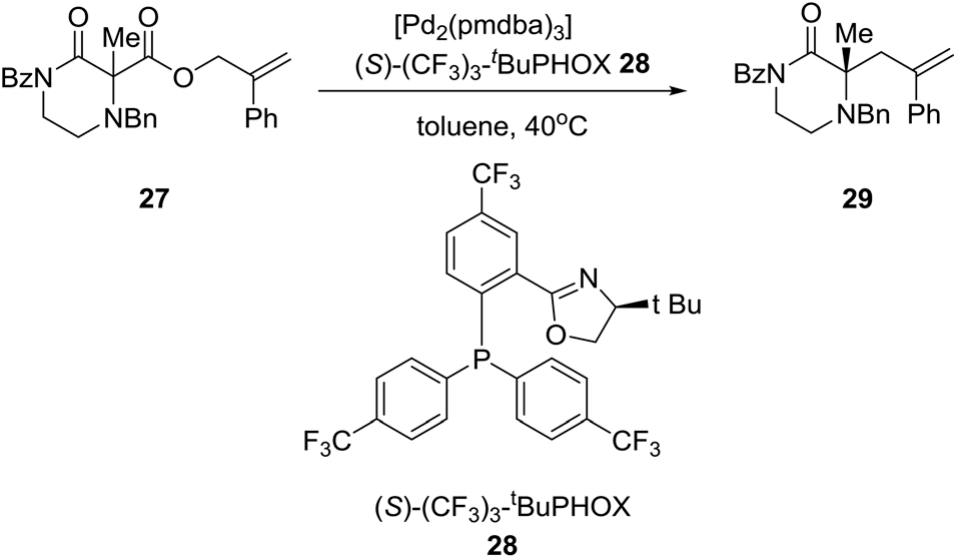



A few years later, the group of Ruijter in Holland described a related process involving an intramolecular Tsuji–Trost reaction of Ugi adducts **30**^23^ for the synthesis of spiro-diketopiperazines **31** [eqn (3), in >90 : 10 enantiomer ratios (er)], which display diverse biological activities, including neuroprotective properties, antiinflammatory activity, and antiproliferative effects against drug-resistant human cancer cell lines.^24^ Obviously, the high er values were achieved by employing the optimized homochiral ligand **32**.3
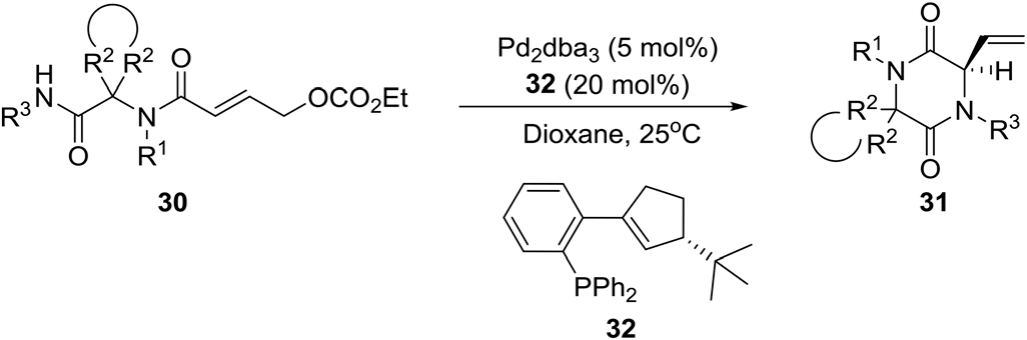



### B. Catalytic diastereoselective approaches

A highly diastereoselective intramolecular palladium-catalyzed hydroamination reaction as a key step in a modular synthesis of 2,6-disubstituted piperazine **33**^25^ has been reported employing an aminoalkene **34** synthesized from a homochiral cyclic sulfamidate **35** (eqn (4)).^26^4
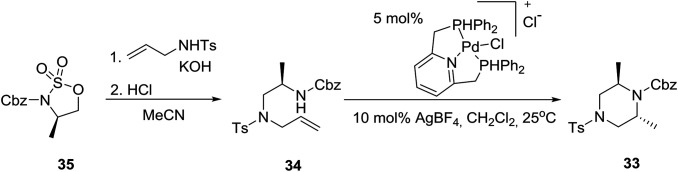



The relative stereochemistry of the piperazine product was determined to be *trans* by single-crystal X-ray diffraction of **33**. Remarkably, the preferred conformation of the piperazine ring was found to be a twist-boat conformation rather than the typical chair conformation. Obviously, this preference is a result of the allylic strain between the methyl substituents and the carbamate protecting group. In the chair conformation, one of the two methyl substituents is forced to adopt an equatorial position adjacent to the carbamate group, which would result in a strong A_1,3_-interaction shown below.^27^ Given the borderline of piperazine derivatives between small molecules and peptides,^23^ the latter concept may find important implications in drug design.
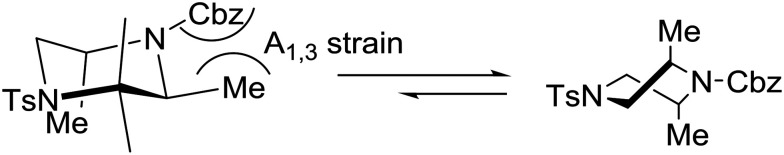


**Scheme 4:**
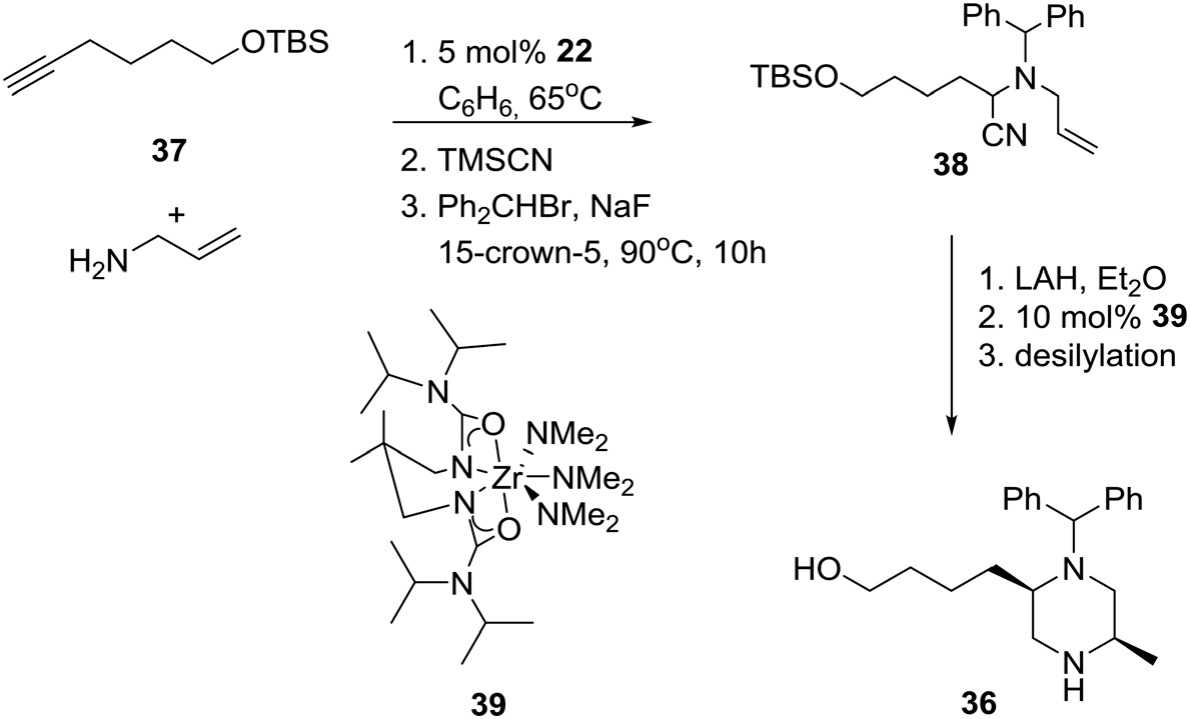
Schafer's catalytic diastereoselective synthesis of piperazines.

Cognizant of their previous results, outlined in the Catalytic enantioselective approaches section, the group of Professor Schafer proceeded to apply their process to the diastereoselective synthesis of 2.5-disubstituted-*N*-benzhydryl-piperazine **36** (Scheme 4) as a selective N-type Ca^2+^ channel blocker aimed at the inhibition of spinal neurotransmitter release and the attenuation of afferent pain signals.^28^

Specifically, anti-Markovnikov regioselective intermolecular hydroamination of terminal alkyne **37** with allylamine, followed by a modified Strecker reaction with trimethylsilyl cyanide and alkylation with benzhydryl bromide, produced α-aminonitrile **38**.^29^ Nitrile reduction of the latter to the corresponding substituted diaminoalkene provided the requisite piperazine **36** in about 15% overall yield after ring-closure using Zr-ureate intramolecular aminoalkene hydroamination catalyst **39** and deprotection of the TBS group. Presumably, the reduced yield of this *N*-benzhydryl substituted piperazine **36** is due to the increased steric bulk of the benzhydryl substituent *versus* the benzyl-substituted amines implicated in the more efficient original synthesis of related *cis*-2,5-disubstituted piperazines.^30^

## Chiral-pool assisted asymmetric syntheses of carbon-substituted piperazines and related heterocycles

### A. Chiral auxiliary based

**Scheme 5:**
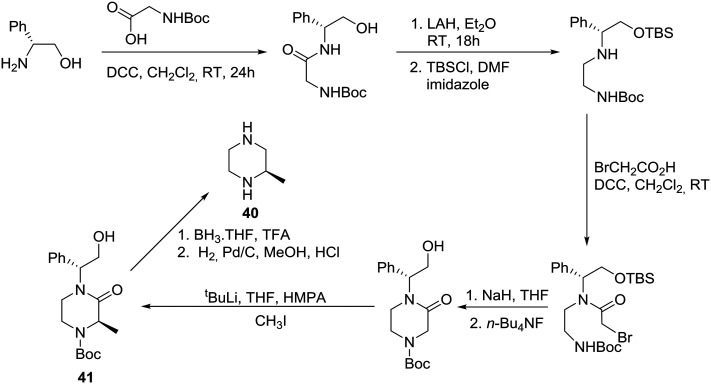
Asymmetric synthesis of 2-methyl piperazine **40**.

The asymmetric synthesis of (*R)*-(+)-2-methypiperazine (**40**) has been reported from R-(–)-phenylglycinol, as the chiral auxiliary, and *N*-Boc glycine *via* the protected 2-oxopiperazine **41** as shown in Scheme 5.^31^

Accordingly, (*R*)-(–)-phenylglycinol was condensed with *N*-Boc glycine in the presence of DCC to give the expected amide, reduction of which followed by protection of the hydroxyl group provided the corresponding silyl ether in 66% overall yield. Selective condensation with bromoacetic acid was achieved in the presence of DCC, followed by cyclization to deliver the requisite 2-oxopiperazine after desilylation. Diastereoselective methylation of the latter proceeded smoothly and furnished 2-oxopiperazine **41** in 80% yield and >90% de. Finally, decarbonylation and debenzylation resulted in the desired (*R*)-(+)-2-methylpiperazine (**40**) in 63% overall yield.

The synthesis of homochiral *cis*- and *trans*-2-phenyl-3-(trifluoromethyl)piperazines involving diastereoselective nucleophilic addition of the Ruppert–Prakash reagent (TMSCF_3_) to homochiral α-amino sulfinylimines, derived from (*R*)-phenylglycinol and bearing Ellman's auxiliary, was reported by a group in Spain.^32^ This methodology allowed an entry to the unknown and stereochemically defined trifluoromethylated piperazines as building blocks in drug discovery due to the noticeable effect of the CF_3_ group on the metabolic stability, basicity, and lipophilicity of the piperazine core.

**Scheme 6:**
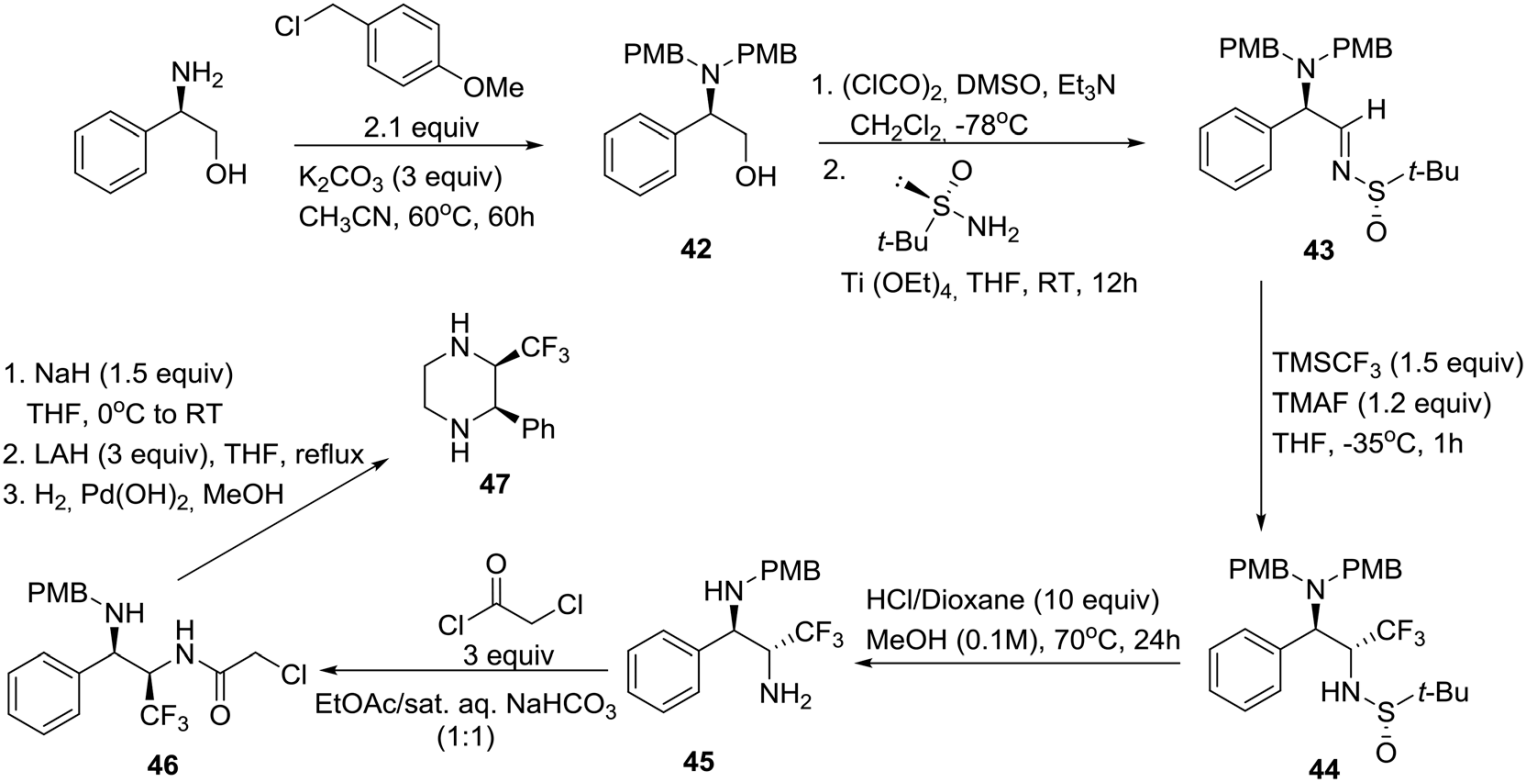
Asymmetric synthesis of *cis*-2-phenyl-3-(trifluoro-methyl)piperazine **47** as a building block for drug discovery.

As shown in Scheme 6, this synthesis began with protection of (*R*)-phenylglycinol as the corresponding bis-*p*-methoxybenzyl (PMB) derivative *R*-**42**. Swern oxidation, followed by condensation of the resulting α-amino aldehyde with (*S*)-(–)-2-methyl-2-propanesulfinamide in the presence of titanium(iv) ethoxide as a dehydrating agent, yielded enantiomerically pure *tert*-butylsulfinimine (*R*,*S*_s_)-**43** ([*α*]25D +121.8). After careful optimization of the key diastereoselective trifluoromethylation, it was found that dropwise addition of TMSCF_3_ to a solution of Ellman's imine **43** and finely ground tetramethylammonium fluoride (TMAF) as an activator of the Ruppert–Prakash reagent in THF at –35 °C produced the protected diamine (*R*,*R*,*S*_s_)-**44** as a single diastereomer. Next, the *tert*-butanesulfinyl group was removed upon exposure to HCl in refluxing methanol in a sealed tube, giving rise to the monoprotected diamine (*R*,*R*)-**45**. Employment of the closed vessel proved essential for the partial unmasking of the benzylic amine, a prerequisite for the subsequent enclosure of both nitrogens into the piperazine ring (Scheme 6). Thus, the reaction of primary amine **45** with 2-chloroacetyl chloride followed by 6-*exo-tet* cyclization of the resulting chloroderivative **46** provided an (*R*,*R*)-oxopiperazine, consecutive reductions of which furnished the desired *cis*-disubstituted (*R*,*R*)-piperazine **47**.^32^ Interestingly, employment of commercially available (*R*)-(+)-2-methyl-2-propanesulfinamide and a slightly different final deprotection sequence yielded the corresponding *trans*-disubstituted (*R*,*S)*-piperazine.^32^

**Scheme 7:**
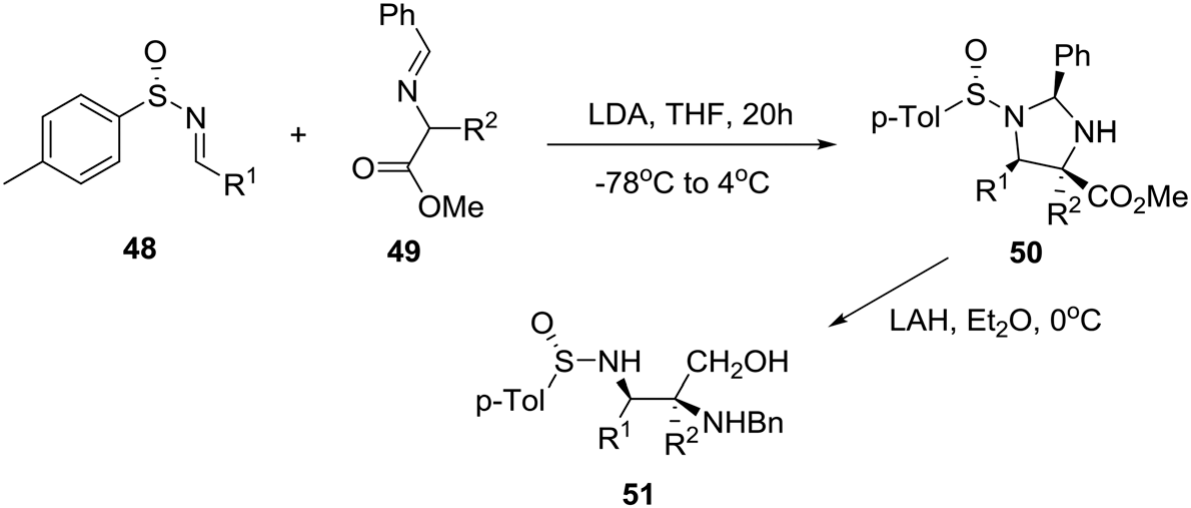
Asymmetric synthesis of enantiopure vicinal diaminoalcohols **51**.

**Scheme 8:**
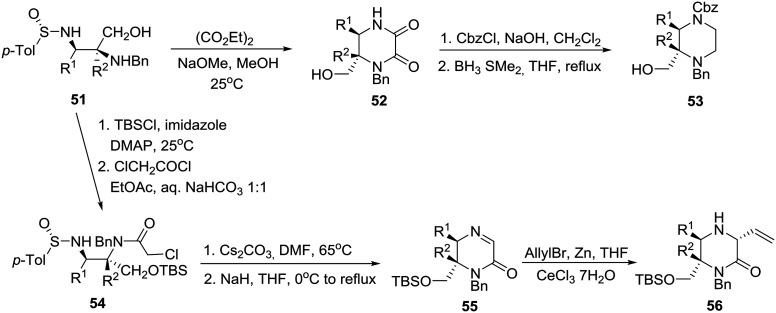
Asymmetric synthesis of disubstituted piperazine **53** and trisubstituted piperazinone **56**.

By exploring previously reported highly diastereoselective [3 + 2] cycloaddition of nonracemic *p*-tolylsulfinimines **48** and iminoesters **49** providing enantiopure imidazolidines **50** and thereby vicinal diaminoalcohols **51** by reduction (Scheme 7),^33^ the group of Viso and coworkers in Spain reported the efficient conversion of these homochiral products into both chiral piperazinones and piperazines as exemplified in Scheme 8.^34^

Specifically, treatment of diaminoalcohol **51** with diethyl oxalate and sodium methoxide in methanol gave rise to 2,3-diketopiperazinone **52**, borane reduction of which furnished enantiopure, orthogonally protected 2,3-disubstituted piperazine **53**. On the other hand, exposure of **51** to TBSCl and chloroacetyl chloride followed by cesium carbonate in DMF at 65 °C and sodium hydride at 0 °C to reflux produced imino ketopiperazine **55** through the intermediacy of **54**. Finally, addition of an allyl organocerium reagent to **55**^35^ gave rise to homochiral trisubstituted piperazinone **56** in 87% yield (Scheme 8).

### B. Chiral amino acid or carbohydrate pool based

Borrowing from the chirality of 4-*O*-trifloxy-2,3-anhydro-ribopypanosides and employing *N*,*N*′-disubstituted ethylenediamines such as **16** (Scheme 1), the group of Voelter in Germany reported the synthesis of chiral tetrasubstituted piperazines of potential pharmacological interest as shown in eqn (5).^36^ Thus, simultaneous nucleophilic attack of the *cis*-oriented epoxy triflate **57** by the two nitrogen atoms of *N*,*N*′-dibenzyl ethylenediamine **16** (Scheme 1) afforded the pyrano-fused tetrasubstituted piperazine **58** in about 80% yield (eqn (5)).5
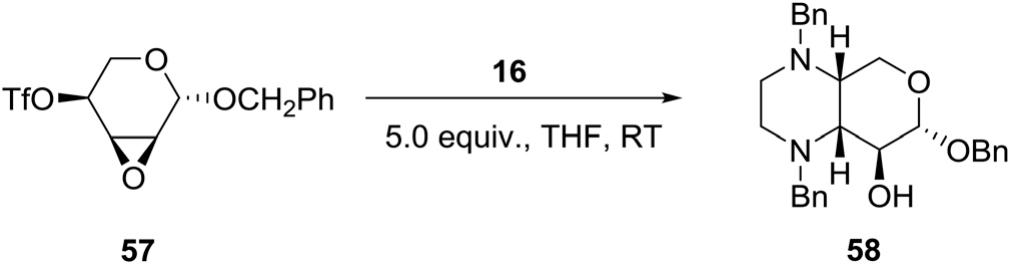



**Scheme 9:**
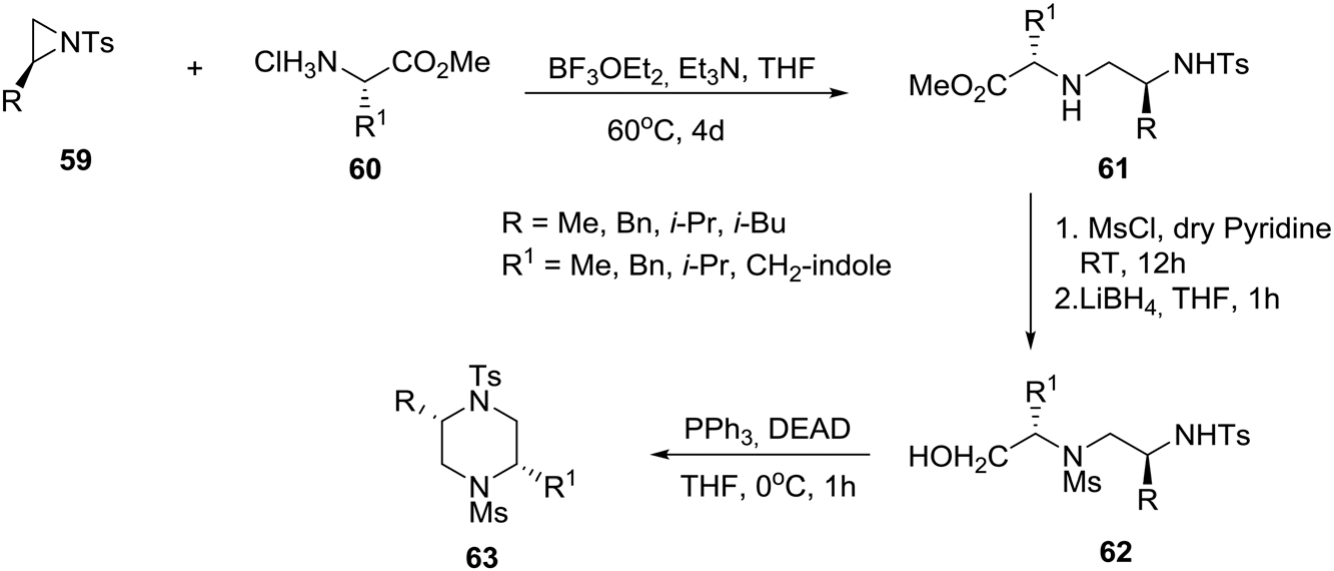
Asymmetric synthesis of *cis*-2,5-disubstituted piperazine **63**.

Exploiting the chiral pool, the group of Panda, at the Central Drug Research Institute, Lucknow in India, reported the synthesis of *cis*-2,5-disubstituted homochiral piperazines by the regioselective ring-opening of natural amino acid-derived chiral aziridines **59** by natural amino acid methyl ester hydrochloride salts **60** (Scheme 9).^37^ In practice, treatment of (*S*)-aziridine **59** with (*S*)-amino acid methyl ester hydrochloride salt **60** in the presence of an equivalent amount of triethylamine and boron trifluoride etherate complex in tetrahydrofuran at 60 °C for four days produced the desired *N*-tosyl diamine **61** in good yields (25–66%, seven examples, Scheme 9). Protection of the secondary amine in **61**, with mesyl chloride followed by reduction of the methyl ester, produced protected diamino-alcohol **62**, intramolecular Mitsunobu cyclization of which furnished the desired *cis*-2,5-disubstituted enantiopure piperazine **63**. A related solid-phase synthesis of selectively *N*-protected homochiral 2,5-disubstituted piperazines has been reported by Franzyk and coworkers in Denmark, featuring a regioselective microwave-assisted aminolysis of resin-bound aziridines with ethanolamine followed by intramolecular Fukuyama–Mitsunobu cyclization.^38^

**Scheme 10:**
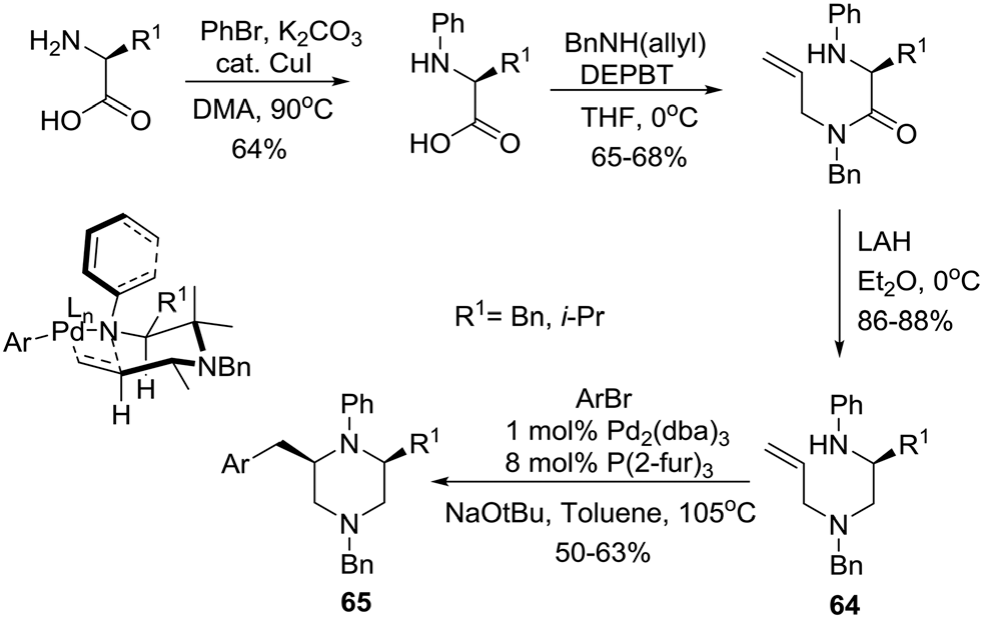
Asymmetric synthesis of *cis*-2,6-disubstituted *N*-aryl piperazines **65**.

Earlier, the group of Wolfe had reported a Pd-catalyzed carboamination approach to the construction of *cis*-2,6-disubstituted *N*-aryl piperazines based on the use of unprotected homochiral amino acids as starting materials.^39^ Specifically, phenylation of the amino group followed by amidation of the carboxyl group with *N*-(benzyl)allylamine employing a 3-(diethoxyphosphoryloxy)-1,2,3-benzotriazin-4(3*H*)-one (DEPBT) reagent^40^ and reduction furnished the desired carboamination substrates **64** that readily underwent Pd-catalyzed transformation with an aryl bromide to furnish *cis*-2,6-disubstituted *N*-aryl piperazines **65** in 50–63% yields and 98–99% ee (Scheme 10). Notably, the use of other reagents such as DCC/HOBT or CDI instead of DEPBT resulted in partial racemization to afford products with 85–90% ee. The working hypothesis for *cis*-piperazine formation involves cyclization *via* a transition state (shown as an insert in Scheme 10) in which the N–Ph group is rotated such as N is pyramidalized. This eliminates the A_1,3_-interaction and allows the pseudoequatorial orientation of R^1^ leading to the *cis*-2,6-disubstituted stereoisomers.^27^

**Scheme 11:**
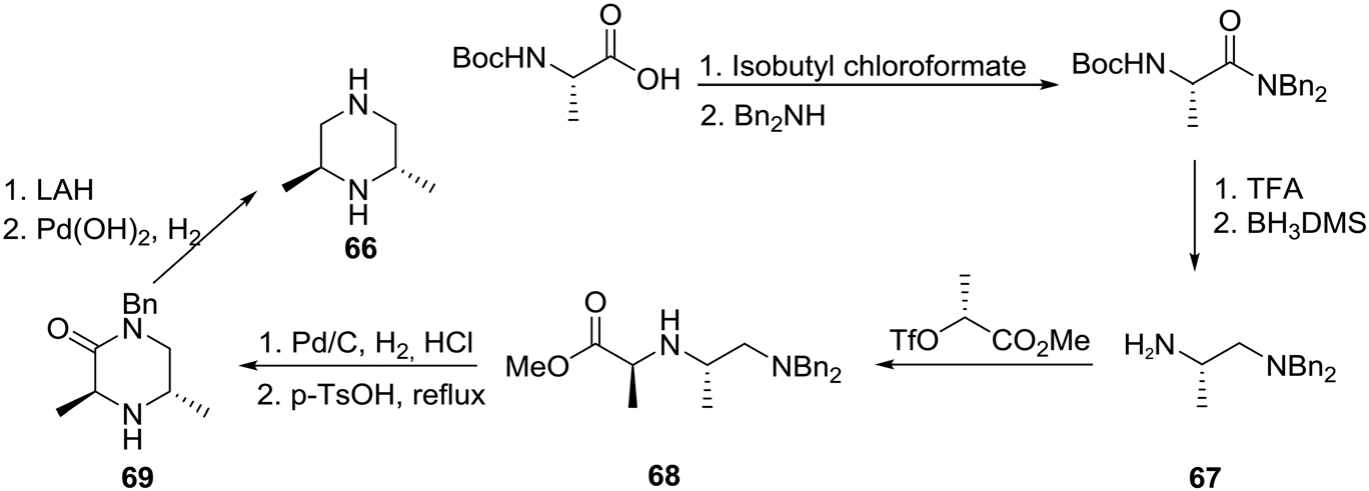
Asymmetric synthesis of 2,6-dimethyl piperazine **66**.

As a part of research in a structure–activity relationship (SAR) study involving the discovery of novel GABA_A_-based anxiolytic agents, a group at the Upjohn Company became interested in preparing analogues incorporating homochiral 2,6-polymethylated piperazines such as **66**. The syntheses involved the employment of an assortment of starting materials readily available from the chiral pool as exemplified in Scheme 11.^41^ Specifically, the conversion of *N-t*-Boc-l-alanine to the corresponding dibenzylamide followed by partial deprotection and reduction gave diamine (+)-**67** in >98% ee. Notably, complete decomplexation of the amine from borane required heating at reflux in aqueous KOH. Next alkylation of **67** with methyl (*R*)-2-[(trifluoromethanesulfonyl)oxy]propionate, generated by the sequential treatment of methyl (*R*)-lactate with trifluoromethanesulfonic anhydride and 2,6-lutidine, proceeded smoothly to furnish ester (–)-**68**, monodebenzylation and cyclization of which provided interesting piperazin-2-one (+)-**69**. Lithium aluminum hydride (LAH) reduction of **69** followed by hydrogenolysis of the remaining benzyl group with Perlman's catalyst gave rise to the desired (2*S*, 6*S*)-2,6-dimethylpiperazine (**66**) in an overall yield of 44% and >98% ee.

Research efforts at Merck around the beginning of this century led to the identification of **MB243**, a potent and selective small-molecule agonist of the MC4R representing one of the five cloned melanocortin receptor subtypes (MC1R–MC5R) which are part of a family of seven-transmembrane G-protein-coupled receptors.^42^
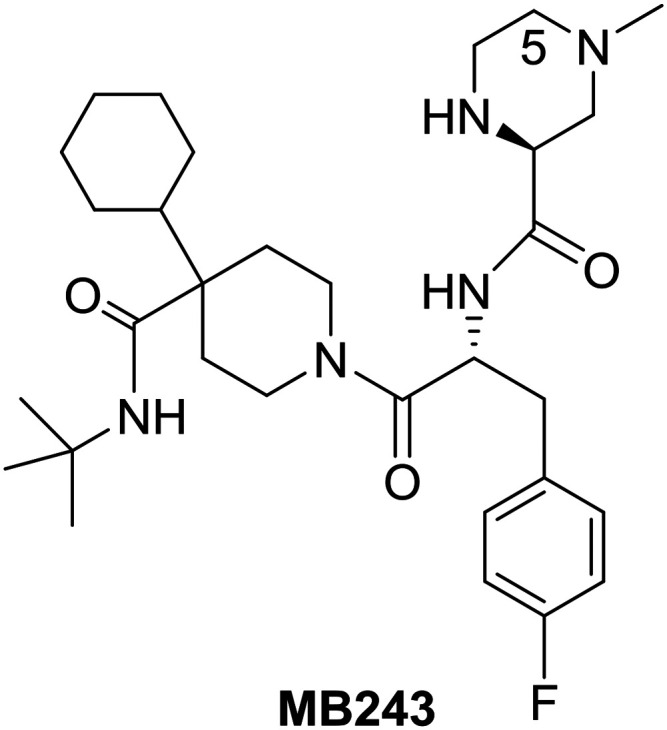


**Scheme 12:**
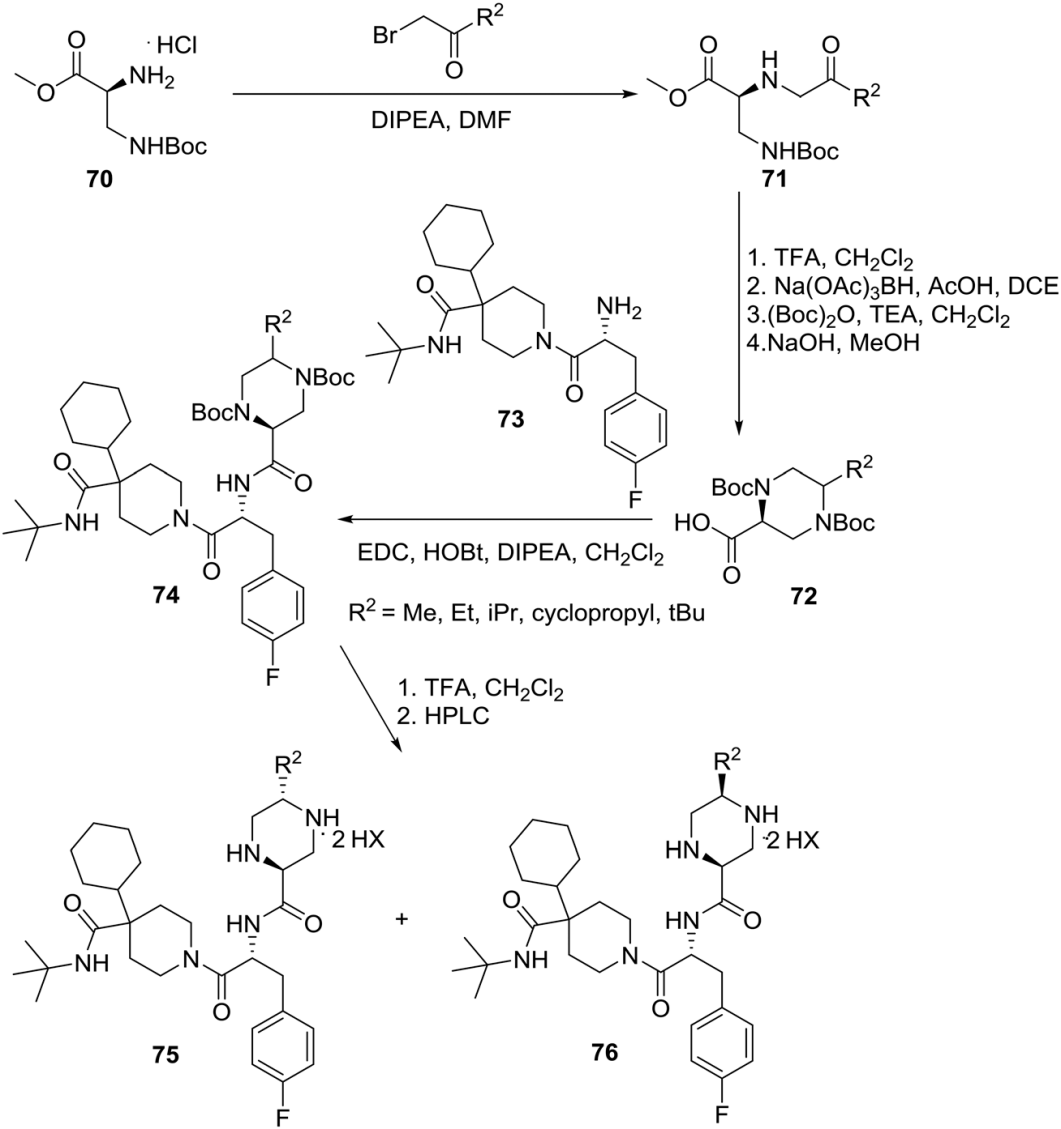
Merck asymmetric synthesis of 2-piperazinecarboxamides as potent and selective MC4R agonists.

Although **MB243** was shown to stimulate erectile activity and reduce food intake in rats, further development of this compound was discontinued due to its potential for bioactivation as seen *in vitro* through extensive covalent binding in rat and human liver microsomes. NMR analysis of the isolated adducts suggested that bioactivation occurred *via* initial oxidation at C-5 on the piperazine ring. Consequently, it was speculated that blocking the site of activation *via* alkylation on the piperazine ring should reduce bioactivation. Accordingly, the 5-alkylated piperazine compounds were prepared in a linear fashion as shown in Scheme 12.^42^

Starting with commercially available methyl *N*-β-Boc-l-α,β-diaminopropionate hydrochloride (**70**), conversion to diamine **71** was achieved *via* alkylation with various substituted α-bromoketones. Removal of the Boc group followed by intramolecular reductive amination provided piperazine of which protection of both secondary amines followed by hydrolysis of the ester yielded the 5-alkylated-2-piperazinecarboxylic acids **72**. Coupling of piperazines **72** with 1-[(2*R*)-2-amino-3-(4-fluoro-phenyl)-1-oxopropyl]-4-cyclohexyl-*N*-(1,1-dimethylethyl)-4-piperidine carboxamide (**73**) and deprotection furnished dipeptides **74** as 2 : 1–10 : 1 mixtures of *trans* : *cis* diastereomers that were separated using preparative reverse-phase HPLC to give *trans*-piperazines **75** and *cis* piperazines **76** as ditrifluoroacetic acid salts (Scheme 12).

An interesting intramolecular [3 + 2]-cycloaddition of an azide to a C–C double bond as a novel approach to piperazines was reported by a group in Russia.^43^ The readily available amino alcohols **77** were converted to their corresponding *N-o*-nitrobenzenesulfonyl derivatives **78**, allylation of which yielded the unstable to polymerization *N*-allyl compounds **79** which were kept in dilute solutions (MeCN) to promote the intramolecular cyclization to the relatively stable triazolines **80**. The target 2-(chloromethyl)piperazines **81** were prepared from **80** by reactions with selected acyl, carbamoyl, and alkyl halides. Notably, the best yields (76–89%) were obtained with CbzCl as shown in eqn (6).6
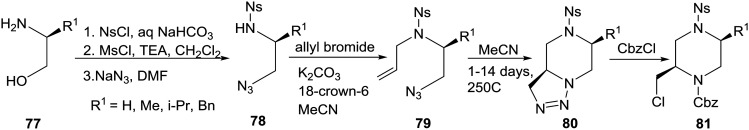



**Scheme 13:**
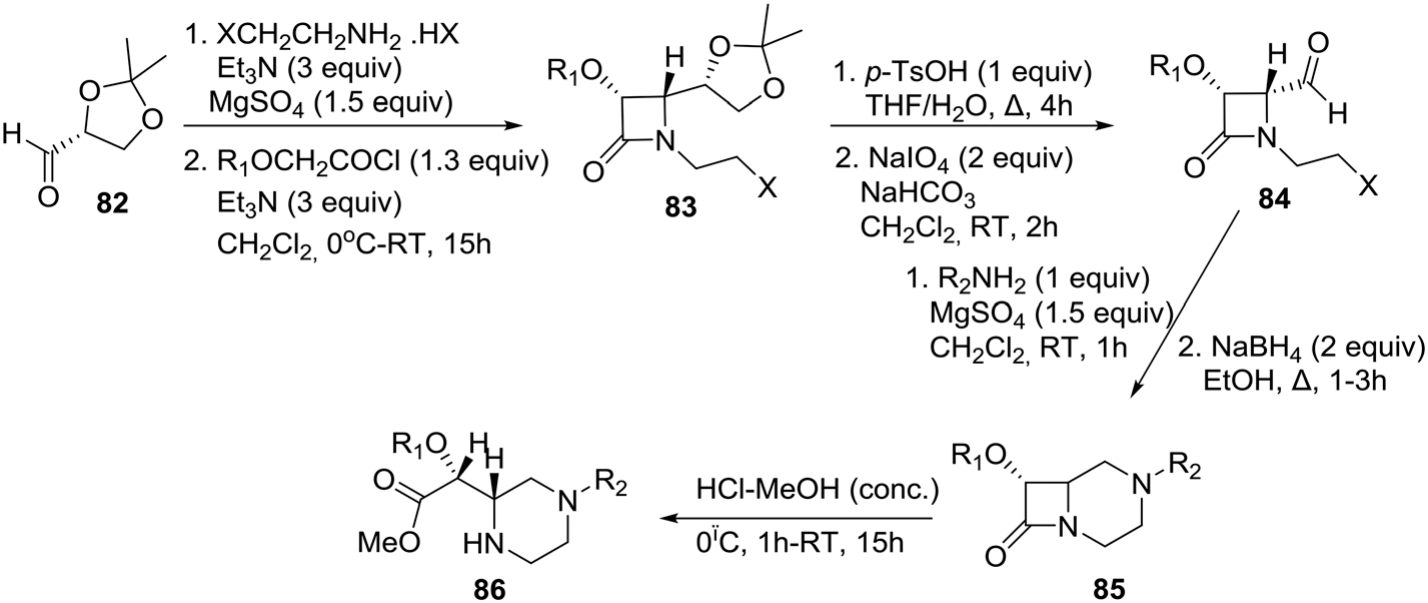
De Kimpe demonstration of the β-lactam synthon methodology toward the asymmetric synthesis of piperazines.

The asymmetric synthesis of 4-formyl-1-(ω-haloalkyl)-β-lactams **84** (Scheme 13) and their transformation into functionalized homochiral piperazines were reported by the group of De Kimpe in Belgium.^44^ The synthesis of chiral 4-formyl-1-(ω-haloalkyl)-β-lactams was performed by means of a slightly modified four-step literature procedure as shown in Scheme 13. (*R*)-Glyceraldehyde acetonide **82** was condensed with 2-chloroethylamine (*in situ* prepared from the corresponding hydrohalide salt using 3 equiv. of Et_3_N) in dichloromethane in the presence of MgSO_4_, and the resulting imine was used as a substrate for the Staudinger reaction using benzyloxy- or methoxyacetyl chloride to construct β-lactam **83** in a highly diastereoselective fashion. The latter azetidin-2-one **83** could be easily converted to the requisite (3*R*, 4*R*)-4-formyl-β-lactam **84** by consecutive hydrolysis and oxidation (Scheme 13).^44^

Intramolecular imination of 4-formyl-1-(2- and 3-haloalkyl)azetidin-2-one **84** upon treatment with 1 equiv. of a primary amine in CH_2_Cl_2_ in the presence of MgSO_4_ and subsequent reduction afforded bicyclic β-lactam **85**, which was then treated with HCl in methanol to provide novel methyl (*R*)-[(*S*)-piperazin-2-yl]acetate **86** in good yield through acid-catalyzed methanolysis of the β-lactam ring. Therefore, the β-lactam synthon methodology,^45^ according to which β-lactams can be employed as useful intermediates for organic synthesis and as synthons for various biologically active compounds, has been amply demonstrated in its homochiral version by this particular example.^46^

An interesting synthetic strategy was described recently, by the group of Panda at the Central Drug Research Institute in India, for the construction of (*S*)-amino acid-derived homochiral *cis*-2,5-disubstituted piperazines. Cu-Catalyzed spontaneous regioselective ring-opening and ring-closing of non-activated *N*-tosyl aziridines as well as Pd-mediated N–C bond formation from *N*-tosyl brominated amino-acid derivatives are the key steps for accessing the disubstituted piperazines.^47^ Although both routes are effective, the Pd-catalyzed process is more favorable than the Cu-catalyzed process, furnishing both symmetrical and unsymmetrical piperazines as illustrated in eqn (7). Mechanistic pathways for the synthesis of symmetrical and unsymmetrical piperazines have been proposed.^47^7
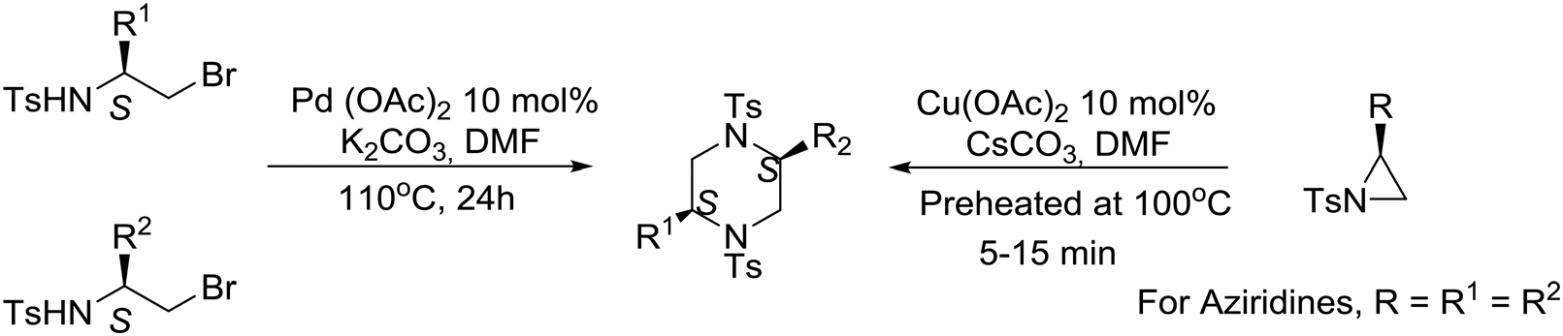



As noted above, a classic method for accessing carbon-substituted piperazines is the reduction of 2,5-diketopiperazines generated *via* peptide cyclodimerization or by the multicomponent Ugi reaction.^23^,^48^ Highlighting their reduction reagent (NaBH_4_/I_2_), the group of Periasamy in India reported the synthesis of the homochiral bicyclic piperazine **87** derived from l-proline (eqn (8)).^49^8
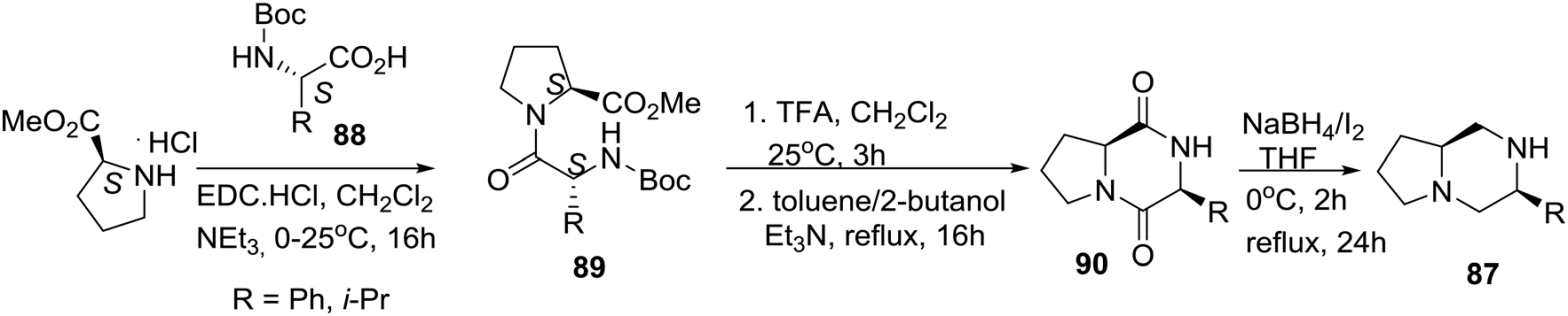



l-Proline methyl ester hydrochloride salt was coupled with *N*-Boc protected amino acid derivatives **88** to prepare dipeptides **89**, which after TFA treatment and cyclization afforded diketopiperazine derivatives **90**. Efficient (68–74% yields) reduction of the latter with the NaBH_4_/I_2_ reagent gave the desired enantiomerically pure bicyclic piperazines **87**.^49^

**Scheme 14:**
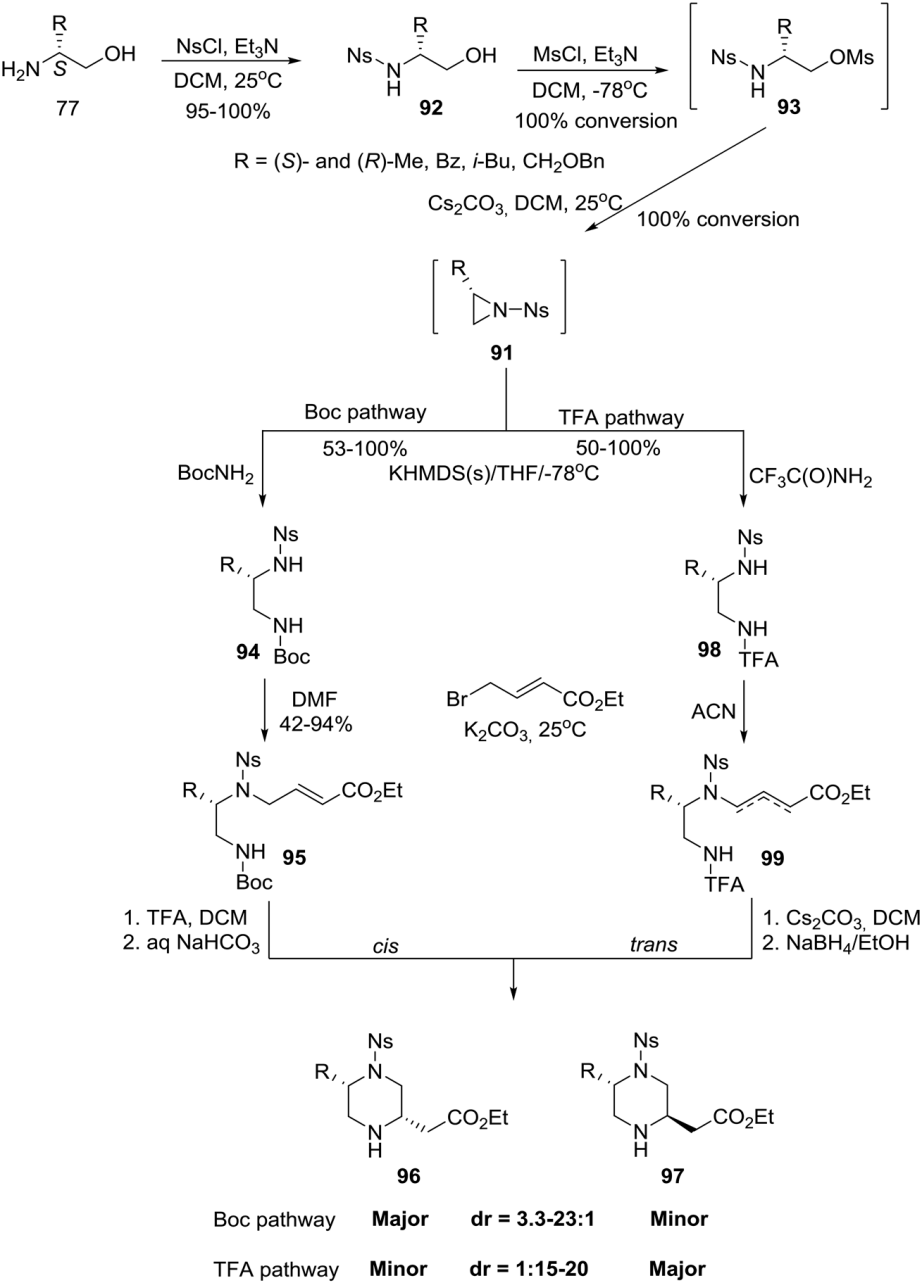
General synthetic scheme for the asymmetric construction of *cis* and *trans* 5-alkyl-substituted piperazine-2-acetic acid esters.

Finally, capitalizing on prior knowledge obtained through studies on the intramolecular aza-Michael reaction,^50^ the group of Santini and Young at Baylor College of Medicine in the U.S. reported the synthesis of homochiral 5- and 6-substituted-piperazine-2-acetic acid esters as intermediates for library production.^51^ In the case of 2,5-disubstituted piperazines, a divergent six-step synthesis was developed in which homochiral amino alcohols **77** (eqn (6)) were transformed, with high diastereoselectivity, into either *cis* or *trans* 5-substituted piperazine-2-acetic acid esters that could be chromatographically rendered diastereomerically homogeneous.51*a* The synthesis proceeded *via* a three step construction of non-isolable *N*-nosyl aziridines **91** which entered two pathways: the *cis* selective Boc pathway and the *trans* selective TFA pathway (Scheme 14).51*a*

Specifically, amino alcohols **77** (eqn (6)) were converted to their *N*-nosylated derivatives **92**, respectively, by exposure to NsCl/TEA in DCM at ambient temperature. The crude products were treated with MsCl/TEA in DCM to give the mesylated intermediates **93** as determined by LC-MS. Mesylates **93** were isolated in crude form by extractive workup and immediately advanced without further characterization. Accordingly, treatment with Cs_2_CO_3_ in DCM effectively generated the *N*-nosyl aziridines **91**, as determined by LC-MS, which were isolated as crude solutions given the instability of the *N*-nosyl aziridine in neat form.51*a* Treatment of aziridines **91** with the preformed potassium salt of BocNH_2_ in THF at –78 °C directly afforded the regiospecifically ring-opened, orthogonally protected diamines **94** that were in turn regioselectively alkylated with ethyl 4-bromocrotonate to obtain the cyclization precursors **95**. Deprotection with TFA/DCM followed by bicarbonate neutralization furnished a mixture of piperazines **96**-*cis* and **97**-*trans* (dr *cis* : *trans* 3.3–23 : 1). Using this route, however, with a dr of >5 : 1, would not enable the authors to obtain enough of the minor *trans* diastereomer to meet their goals. They, therefore, surmised that having a substituted nucleophilic nitrogen would increase the steric demand of the aza-Michael donor, a consequence that might alter the diastereoselectivity of the cyclization. Thus, they pursued a base-promoted anionic cyclization of a carbamate, namely the TFA pathway: treatment of aziridines **91** with the preformed potassium salt of CF_3_C(O)NH_2_ afforded the differentially substituted diamines **98**. Alkylation of diamines **98** with ethyl 4-bromocrotonate proceeded more cleanly in ACN (acetonitrile) than in DMF (dimethylformamide) and produced a 1.5 : 1 [{2,3} : {3,4}] mixture of the olefin isomers **99** which were individually advanced through the base-promoted cyclization. Gratifyingly, piperazines **97**-*trans* were obtained after removal of the TFA group (Scheme 14).

**Scheme 15:**
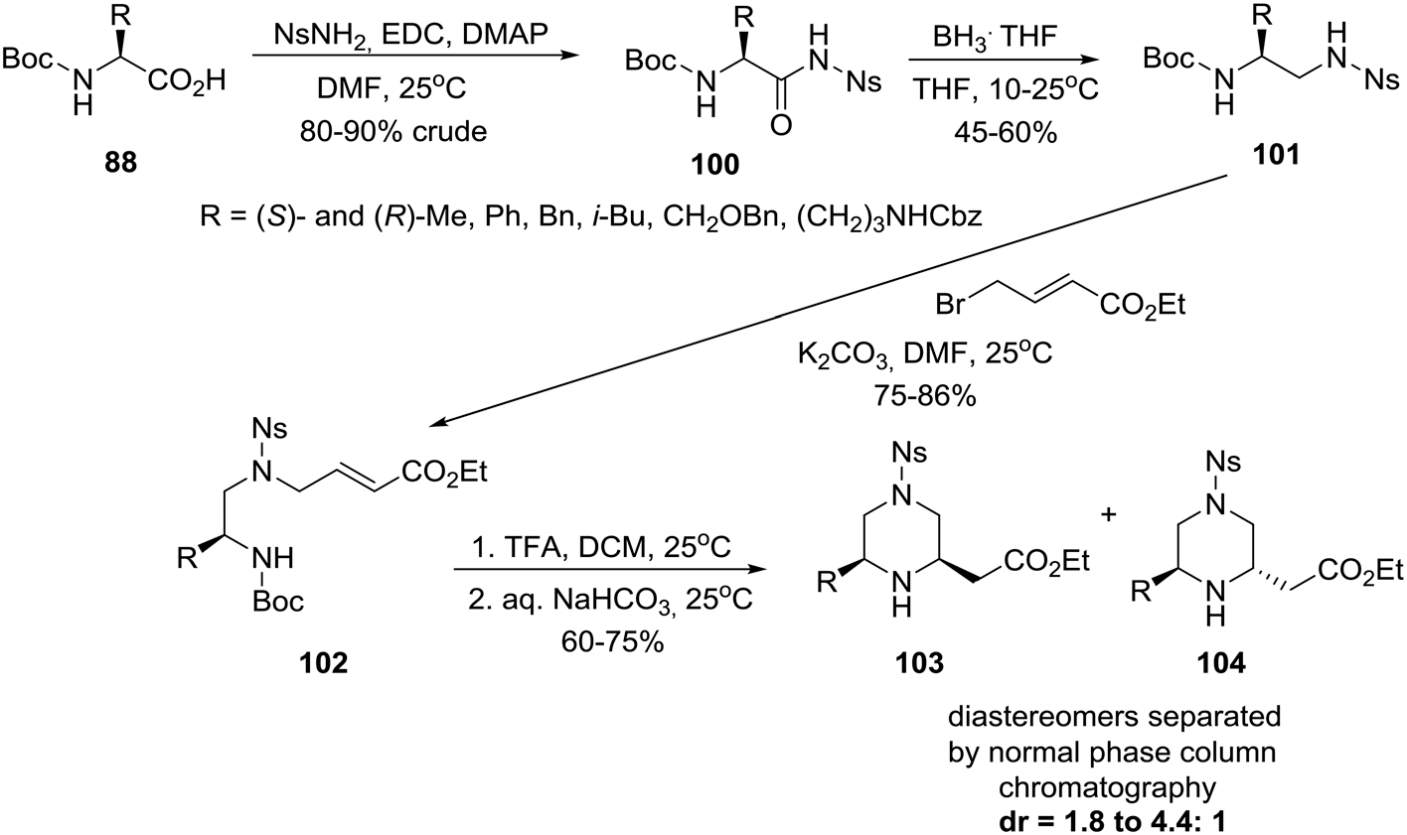
Asymmetric construction of *cis* and *trans* 6-alkyl-substituted piperazine-2-acetic acid esters.

On the other hand, an efficient four-step synthesis allowed transformation of six homochiral amino acids into 6-substituted piperazine-2-acetic acid esters as diastereomeric mixtures of which *cis* and *trans* products could be chromatographically separated (Scheme 15).51*b*

Thus, homochiral amino acids **88** (eqn (8)) were converted to nosylamides **100** by the action of EDC/DMAP in the presence of NsNH_2_. Pleasingly, the nosyl amides **100**, recovered in crude form by extractive workup, could be directly reduced by a BH_3_THF complex to the differentially protected diamines **101**, alkylation of which with ethyl 4-bromo-*E*-crotonate, as described in Scheme 15, gave acrylates **102**. Removal of the *N*-Boc protecting group with TFA/DCM followed by neutralization cleanly afforded a mixture of piperazines from which diastereomers **103***cis* and **104***trans* were isolated by chromatography.51*b*

**Scheme 16:**
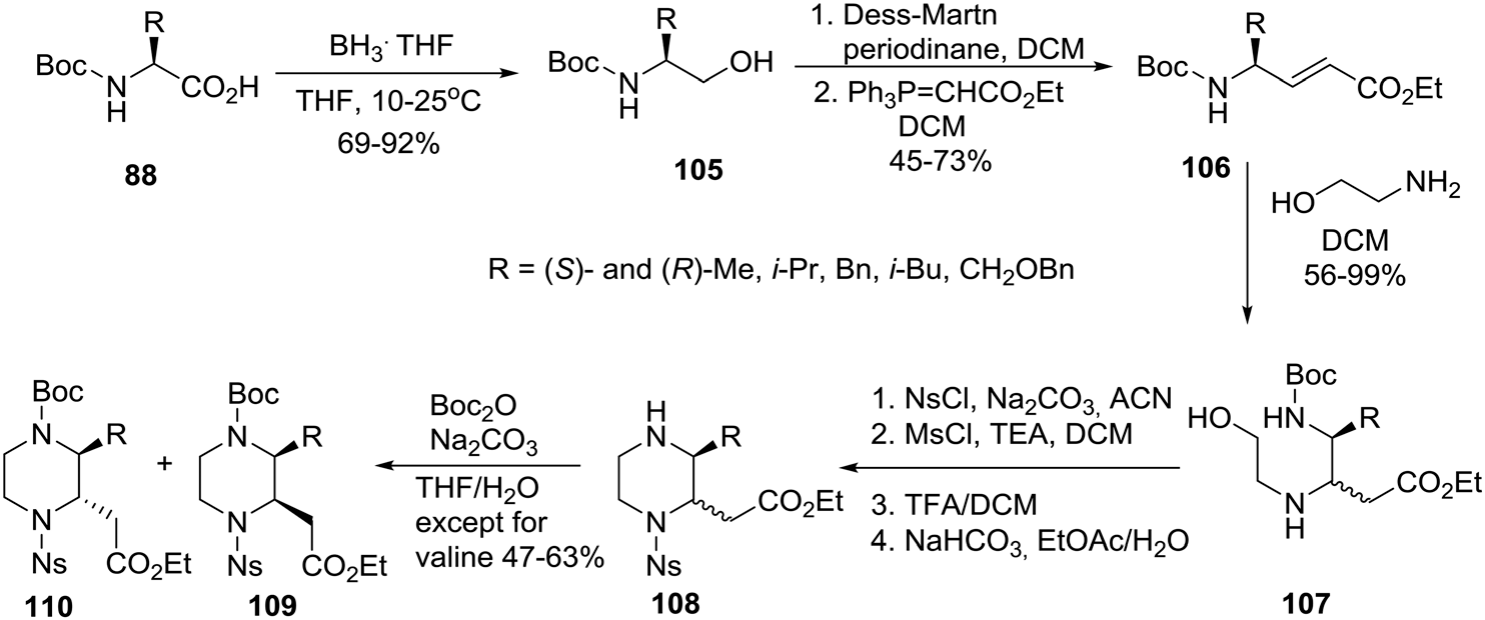
Asymmetric synthesis of *cis* and *trans* 3-alkyl-substituted piperazine-2-acetic acid esters.

To complete the series, for the synthesis of homochiral 3-substituted-piperazine-2-acetic acid esters as intermediates for library production, the group of Santini and Young at Baylor College of Medicine adopted an “inter” retrosynthetic strategy entailing the formation of a second stereocenter by an intermolecular aza-Michael reaction of ethanolamine to an acrylate also derived from a homochiral amino acid **88** (Scheme 16).^52^

The homochiral *N*-Boc amino acids **88** were reduced to their corresponding alcohols **105** by exposure to the BH_3_THF complex. Subsequent oxidation using the Dess–Martin conditions generated the respective aldehydes (not shown, no loss of enantiomeric purity) that were immediately treated with (carbethoxymethylene)triphenylphosphorane to afford the acrylates **106** all having a *trans* geometry. The latter were treated with ethanolamine (4 equiv.) to bring about the aza-Michael reaction which occurred at ambient temperature with modest diastereoselectivity affording mixtures of diastereomers **107**, protection of which with NsCl and reaction with MsCl/TEA gave the *O*-mesylated intermediates (not shown) that were deprotected with TFA to furnish the piperazines **108** after neutralization. The separation of some piperazines proved to be difficult at this point, but Boc reprotection of the nitrogen at the 4-position rendered the remaining diastereomers **109***cis* and **110***trans* separable by normal-phase column chromatography.^52^ In the *trans* products, substituents at C_2_ and C_3_ are diaxial to minimize the allylic 1,3-strain with the nosyl amide as well as to avoid steric clashes that would occur between adjacent diequatorial groups.^27^ The strategic preparation and separation of diastereomeric mixtures proved to be key to the efficient implementation of this systematic chemical diversity (SCD) strategy.^51^,^52^

## Conclusion and outlook

As mentioned in the Introduction section, despite the ubiquity of piperazine-containing therapeutics, the structural, regiochemical, and stereochemical diversity of these compounds along the piperazine's carbon framework is remarkably limited. Notwithstanding the recent progress on asymmetric synthesis of piperazines described in this review, the development of a general method for the enantioselective construction of carbon-substituted piperazine cores has yet to be achieved. This observation provokes the query of whether stereochemically diverse piperazines represent a class of inconsequential compounds or whether current synthetic limitations are hindering access to these stereochemically more elaborate targets.^53^ Nevertheless, efforts toward the development of a general, catalytic enantioselective synthetic approach for carbon-substituted piperazines are underway in this laboratory.

## Conflicts of interest

There is no conflict of interest to declare.
